# In Silico and In Vitro Screening of 50 Curcumin Compounds as EGFR and NF-κB Inhibitors

**DOI:** 10.3390/ijms23073966

**Published:** 2022-04-02

**Authors:** Mohamed E. M. Saeed, Rümeysa Yücer, Mona Dawood, Mohamed-Elamir F. Hegazy, Assia Drif, Edna Ooko, Onat Kadioglu, Ean-Jeong Seo, Fadhil S. Kamounah, Salam J. Titinchi, Beatrice Bachmeier, Thomas Efferth

**Affiliations:** 1 Department of Pharmaceutical Biology, Institute of Pharmaceutical and Biomedical Sciences, Johannes Gutenberg University, Staudinger Weg 5, 55128 Mainz, Germany; saeed@uni-mainz.de (M.E.M.S.); ryuecer@students.uni-mainz.de (R.Y.); modawood@uni-mainz.de (M.D.); hegazy@uni-mainz.de (M.-E.F.H.); drif@uni-mainz.de (A.D.); ooko@uni-mainz.de (E.O.); kadioglu@uni-mainz.de (O.K.); seo@uni-mainz.de (E.-J.S.); 2 Department of Pharmacognosy, Hamidiye Faculty of Pharmacy, University of Health Sciences, Üsküdar, Istanbul 34668, Turkey; 3 Department of Pharmacognosy and Phytochemistry, Faculty of Pharmacy, Bezmialem Vakif University, Istanbul 34093, Turkey; 4 Faculty of Medical Laboratory Sciences, Al-Neelain University, Khartoum 11121, Sudan; 5 Chemistry of Medicinal Plants Department, National Research Centre, 33 El-Bohouth St., Dokki, Giza 12622, Egypt; 6 Department of Chemistry, University of Copenhagen, Universitetsparken 5, 2100 Copenhagen, Denmark; fadil@chem.ku.dk; 7 Department of Chemistry, University of the Western Cape, P/B X17, Bellville, Cape Town 7535, South Africa; titinchi@uni-mainz.de; 8 Institute of Pharmaceutical Biology, Goethe University Frankfurt, 60438 Frankfurt am Main, Germany; b.bachmeier@em.uni-frankfurt.de

**Keywords:** bioinformatics, cancer, natural products, phytochemicals, synthetic derivatives, virtual drug screening

## Abstract

The improvement of cancer chemotherapy remains a major challenge, and thus new drugs are urgently required to develop new treatment regimes. Curcumin, a polyphenolic antioxidant derived from the rhizome of turmeric (*Curcuma longa* L.), has undergone extensive preclinical investigations and, thereby, displayed remarkable efficacy in vitro and in vivo against cancer and other disorders. However, pharmacological limitations of curcumin stimulated the synthesis of numerous novel curcumin analogs, which need to be evaluated for their therapeutic potential. In the present study, we calculated the binding affinities of 50 curcumin derivatives to known cancer-related target proteins of curcumin, i.e., epidermal growth factor receptor (EGFR) and nuclear factor κB (NF-κB) by using a molecular docking approach. The binding energies for EGFR were in a range of −12.12 (±0.21) to −7.34 (±0.07) kcal/mol and those for NF-κB ranged from −12.97 (±0.47) to −6.24 (±0.06) kcal/mol, indicating similar binding affinities of the curcumin compounds for both target proteins. The predicted receptor-ligand binding constants for EGFR and curcumin derivatives were in a range of 0.00013 (±0.00006) to 3.45 (±0.10) µM and for NF-κB in a range of 0.0004 (±0.0003) to 10.05 (±4.03) µM, indicating that the receptor-ligand binding was more stable for EGFR than for NF-κB. Twenty out of 50 curcumin compounds showed binding energies to NF-κB smaller than −10 kcal/mol, while curcumin as a lead compound revealed free binding energies of >−10 kcal/mol. Comparable data were obtained for EGFR: 15 out of 50 curcumin compounds were bound to EGFR with free binding energies of <−10 kcal/mol, while the binding affinity of curcumin itself was >−10 kcal/mol. This indicates that the derivatization of curcumin may indeed be a promising strategy to improve targe specificity and to obtain more effective anticancer drug candidates. The in silico results have been exemplarily validated using microscale thermophoresis. The bioactivity has been further investigated by using resazurin cell viability assay, lactate dehydrogenase assay, flow cytometric measurement of reactive oxygen species, and annexin V/propidium iodide assay. In conclusion, molecular docking represents a valuable approach to facilitate and speed up the identification of novel targeted curcumin-based drugs to treat cancer.

## 1. Introduction

Cancer is currently one of the leading causes of death worldwide. Incidence and death rates are increasing for several types of cancer [1,2]. More than 21 million cancer incidences have been predicted to occur in the year 2025 (https://www.statista.com/statistics/1031316/new-cancer-cases-forecast-worldwide/; accessed on 1 March 2022). Many advances have been made in the early diagnosis and treatment of cancer. However, lowering cancer mortality rates still remains a vital challenge [3]. Cancer chemotherapy involves using natural or synthetic chemicals to prevent or suppress cancer growth. As a matter of fact, more than half of all available anticancer drugs are derivatives of natural products or are compounds that mimic the modes of action of natural products [4]. The advantages of phytochemicals are their lower toxicity profiles and their ability to target multiple signaling pathways that prevent the rapid development of drug resistance [5,6,7]. A number of phytochemicals are known to address cancer-related target proteins, such as the epidermal growth factor receptor (EGFR) [8,9,10,11] or the nuclear factor-kappa B (NF-κB) [12,13,14] and others. 

Curcumin is a naturally occurring compound derived from the rhizomes of *Curcuma longa* L. As a member of the ginger family, it has been commonly used as a spice for food preservation as well as in folk medicine. It possesses a wide array of functional characteristics, including antioxidant and anti-inflammatory [15] as well as antiviral, antibacterial, and antifungal properties [16]. Curcumin has been extensively investigated for its cellular and molecular modes of action against cancer [17,18], diabetes [19], neurological ailments [20], and osteoarthritis [21], and even entered several clinical trials [22,23]. Curcumin inhibits cancer cell proliferation [24,25], DNA repair along the p53-p21/GADD45A-cyclin/CDK-Rb/E2F-DNMT1 axis [26,27], metastasis by the NF-κB/c-JUN/MMP pathway [28], and the CXC-chemokine/NF-κB signaling pathway [29,30,31] as well as angiogenesis by the protein kinase C/NF-κB/AP-1 pathway [32,33]. EGFR is upregulated in several tumor types, including lung and colorectal tumors making EGFR an exquisite therapeutic target. Curcumin targets EGFR in lung cancer [34,35] and colorectal carcinoma [36,37] types leading to tumor cell killing. The transcription factor NF-κB is targeted by curcumin in a wide range of tumor types, including leukemia and lymphoma [38,39,40,41,42].

In contrast to conventional anticancer drugs that often exert severe side effects such as myelosuppression, mucositis, alopecia, nausea, vomiting, and others, curcumin displays only minimal toxicity [43,44,45]. However, the poor bioavailability of curcumin represents a major disadvantage for its clinical application [46]. Many efforts have been undertaken to improve its bioavailability using a variety of approaches, including innovative drug delivery systems (nanoparticles, liposomes, phospholipids, etc.) as well as the development of novel synthetic curcumin derivatives [47,48,49,50,51]. By synthesizing chemical libraries of curcumin derivatives and subjecting them to biological scrutiny, compounds with improved pharmacological features may be yielded.

Our study focuses on curcumin and a total of 50 curcumin compounds that were either reported by us [17] or mined in the PubChem database (https://pubchem.ncbi.nlm.nih.gov; accessed on 31 October 2021). We attempted to predict their activity using an in silico molecular docking approach. For this reason, we calculated the binding energies of these derivatives to two cancer-related proteins, i.e., EGFR and NF-κB. These proteins have been previously described as target proteins of curcumin [52]. 

The epidermal growth factor receptor (EGFR) is a tyrosine kinase in the cell membrane of many tumor types. This transmembrane receptor belongs to a gene family with three other members (HER2-4). The binding of extracellular ligands, i.e., epidermal growth factor (EGF) and transforming growth factor (TGFα), leads to dimerization, autophosphorylation, and downstream activation of signal transduction pathways. This ultimately leads to carcinogenesis, cell growth, metastasis, and inhibition of apoptosis [53], as well as the development of resistance to cytotoxic chemotherapy and radiotherapy [54]. Targeted therapies with small molecules (e.g., erlotinib, gefitinib, afatinib) or monoclonal antibodies (e.g., cetuximab, panitumumab) have significantly improved the treatment outcome of innumerable patients [55]. However, resistance to these targeted drugs has been emerging [56,57]. Thus, the search for new EGFR inhibitors has to continue [58]. In this context, phytochemicals gained interest as novel lead compounds [59]. 

The nuclear factor ‘kappa-light-chain-enhancer’ of activated B-cells (NF-κB) is a transcription factor that binds to specific DNA sequences (the 10 bp long κB motif) and thereby regulates the expression of downstream genes. This primarily involves genes that control the immune response, inflammation, cell proliferation, and apoptosis [60,61]. Since NF-κB activation is important for cancer development and progression [62], this transcription factor has become a target molecule for new drug development [63,64]. Phytochemicals represent an important reservoir for the identification of NF-κB inhibitors in cancer [65,66,67]. Interestingly, EGFR and NF-κB cooperate in tumor cells, amplifying their oncogenic signals [68]. Therefore, it is particularly useful to investigate inhibitors that inhibit both cancer-related proteins simultaneously to achieve a more effective antitumor effect. 

The concept of the present investigation was to identify curcumin derivatives with better binding affinities to EGFR and NF-κB to improve tumor specificity and reduce side effects on normal organs. To validate the molecular docking data, we exemplarily tested the inhibitory effect of curcumin and two of the derivatives investigated by western blot experiments with these four proteins towards EGFR and NF-κB. Our results support the concept that novel synthetic curcumin derivatives with improved specificity to important cancer-related targets could be identified by combined in silico–in vitro drug screening approaches. 

## 2. Results

### 2.1. Molecular Docking 

We first performed molecular docking of 50 curcumin compounds (Figure S1) mined from the PubChem database (https://pubchem.ncbi.nlm.nih.gov/; accessed on 31 October 2021) against EGFR and NF-κB. We intended to predict the potential activity of the synthetic derivatives by calculating their in silico binding activities to these proteins. It is noteworthy that the synthetic curcumin derivatives showed low binding energy values (i.e., higher affinities) to both target proteins.

**Figure 1:**
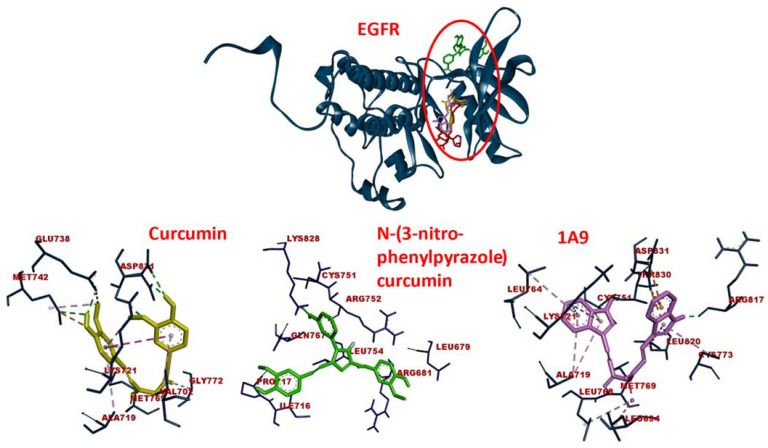
Molecular docking of curcumin-type compounds to EGFR. Top: The compounds were bound to the same domain of EGFR. Bottom: curcumin, *N*-(3-nitrophenylpyrazole) curcumin, and the derivative 1A9 were bound to different amino acids in this domain. The red circle indicates the binding site of the three compounds.

The binding energies for EGFR were in a range of −12.12 (±0.21) to −7.34 (±0.07) kcal/mol and those for NF-κB ranged from −12.97 (±0.47) to −6.24 (±0.06) kcal/mol, indicating similar binding affinities of the curcumin compounds for both target proteins (Table 1). Molecular dockings of curcumin and two selected curcumin derivatives to EGFR are shown in Figure 1. The three compounds were bound to the same domain but with different amino acids within this pharmacophore.

**Figure 2:**
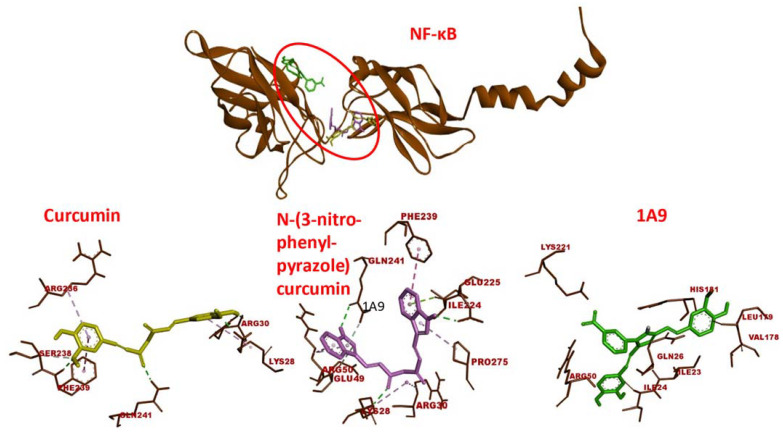
Molecular docking of curcumin-type compounds to NF-κB. Top: The compounds were bound to the same domain of EGFR. Bottom: curcumin, *N*-(3-nitrophenylpyrazole) curcumin, and the derivative 1A9 were bound to different amino acids in this domain. The red circle indicates the binding site of the three compounds.

The predicted receptor-ligand binding constants for EGFR and curcumin derivatives were in a range of 0.00013 (±00.0006) to 3.45 (±0.10) µM and for NF-κB in a range of 0.0004 (±0.0003) to 10.05 (±4.03) µM, indicating that the receptor-ligand binding was more stable for EGFR than for NF-κB (Table 1). Molecular dockings of curcumin and two selected curcumin derivatives to NF-κB are shown in Figure 2. Like EGFR, the three compounds were bound to the same domain of NF-κB.

**Figure 3:**
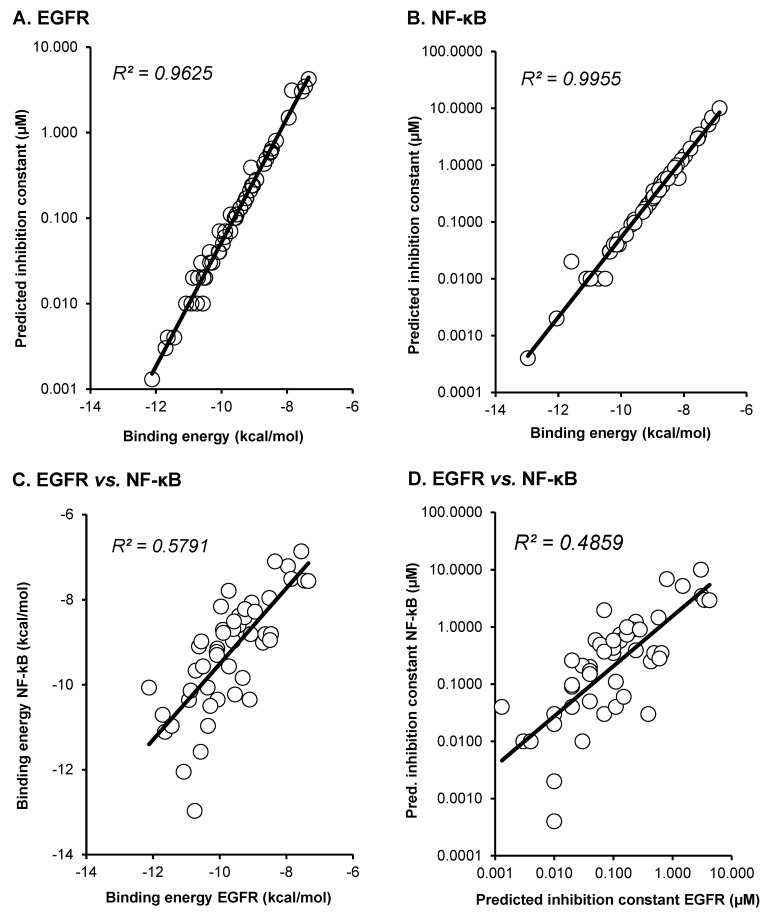
Correlation of binding energies (kcal/mol) and predicted inhibition constants (pKi, µM) of 50 curcumin compounds were calculated using the Pearson correlation test. Correlation of binding energies and pKi values for (**A**) EGFR and (**B**) NF-κB. Correlation of (**C**) binding energies or (**D**) pKi values between EGFR and NF-κB.

As a next step, we correlated the binding energies of the compounds for EGFR and NF-κB. By using Pearson correlation test, we found statistically significant relationships between binding energies and predicted inhibition constants of EGFR (*p* = 2.73 × 10^−9^; *r* = 0.715) and of (*p* = 4.39 × 10^−7^; *r* = 0.631) as well as of binding energies between EGFR and NF-κB (*p* = 4.32 × 10^−5^; *r* = 0.526) and predicted binding energies between both proteins (*p* = 2.53 × 10^−8^; *r* = 0.682). These results indicate that there may be a relationship between the “druggability” of the compounds being EGFR or NF-κB inhibitors. Compounds that were better bound to EGFR also showed a significant relationship to better bind to NF-κB and vice versa (Figure 3). 

### 2.2. Microscale Thermophoresis

**Figure 4:**
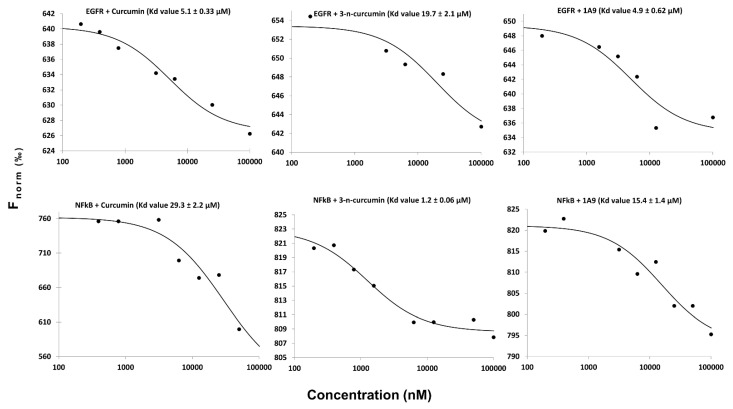
Analysis of the interaction between curcumin derivatives with recombinant EGFR and NF-κB by microscale thermophoresis (MST). The recombinant proteins were used at a concentration of 200 nM, while the concentration of curcumin, *N*-(3-nitrophenylpyrazole) curcumin, and the curcumin derivative 1A9 ranged from 100 to 100,000 nM. The migration of the fluorescent proteins was determined upon local heating using a Monolith NT.115^Pico^ with 40% LED power and 80% MST power for EGFR and with 20% LED power and 20% MST power for NF-κB at room temperature.

To verify the in silico predictions, we performed microscale thermophoresis. This is a biophysical assay to study the interactions between chemical ligands and their target proteins. For this reason, we used recombinant EGFR and NF-κB and assayed them with curcumin, *N*-(3-nitrophenylpyrazole) curcumin, and curcumin derivative 1A9. The equilibrium constants (K_D_) indicate that the three curcumin-type compounds were bound to EGFR and NF-κB (Figure 4). 

### 2.3. Resazurin Assay

**Figure 5:**
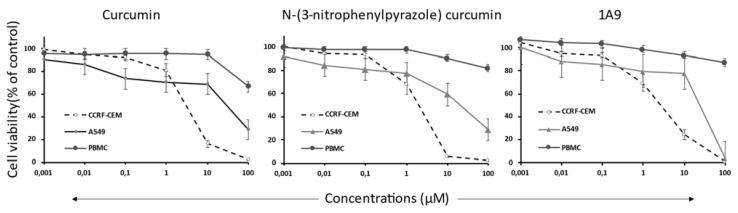
Cell viability dose-response curves of CCRF-CEM leukemia cells, A549 lung carcinoma cells, and healthy peripheral blood mononuclear cells treated with curcumin, 1A9, and *N*-(3-nitrophenylpyrazole) curcumin as determined by the resazurin assay. Cells were incubated with concentrations from 10^−3^ to 100 µM and incubated at 37 °C for 72 h. DMSO was used as vehicle control. Mean ± SD of three independent measurements.

To study the effect of selected compounds on cell viability, we treated human CCRF-CEM leukemia and human A549 lung cancer cells with curcumin, *N*-(3-nitrophenylpyrazole) curcumin, and the curcumin derivative 1A9. CCRF-CEM and A549 cells were chosen as examples of hematopoietic and solid tumor cells. Peripheral blood mononuclear cells (PBMCs) were isolated from a healthy subject to compare the inhibitory effects of the curcumin compounds between tumor and normal cells. As shown in Figure 5, all three compounds inhibited the viability of CCRF-CEM and A549 cells in a dose-dependent manner, while normal PBMCs were not or only minimally inhibited. The dose-response curves were taken to calculate the 50% inhibition concentrations (IC_50_). CCRF-CEM leukemia cells were about one order of magnitude more sensitive to the compounds than A549 cells and *N*-(3-nitrophenylpyrazole) curcumin revealed a higher inhibitory activity than the other two compounds (Table 2). 

### 2.4. LDH Assay

**Figure 6:**
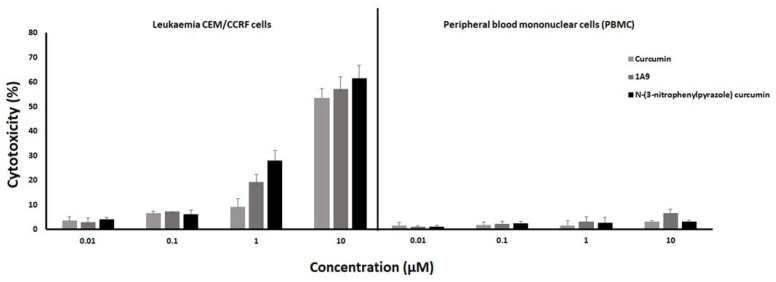
Cytotoxicity in CCRF-CEM leukemia cells (left) and peripheral blood mononuclear cells (PBMCs) of a healthy donor (right) by curcumin, 1A9, and *N*-(3-nitrophenylpyrazole) curcumin as determined by the release of lactate dehydrogenase. Cells were incubated with concentrations from 0.01 to 10 µM and incubated at 37 °C for 48 h. DMSO was used as vehicle control. Mean ± SD of three independent measurements.

To assess the cytotoxicity, the LDH cell death assay was performed that measures the release of LDH from cells due to membrane damage. As shown in Figure 6, all three curcumin-type compounds led to a dose-dependent LDH release in a concentration range of 0.01 to 10 µM in CCRF-CEM leukemia cells. Only negligible LDH release was found in healthy peripheral blood mononuclear cells, indicating tumor-specific cytotoxic effects of the three curcumin compounds.

### 2.5. ROS Assay 

**Figure 7:**
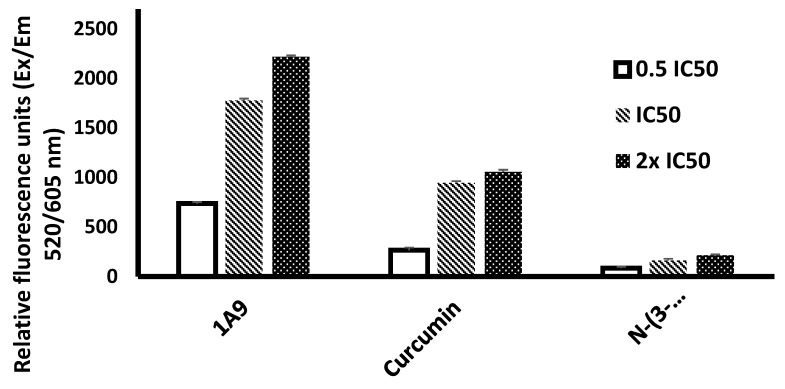
Generation of reactive oxygen species in CCRF-CEM cells by curcumin, *N*-(3-nitrophenylpyrazole) curcumin, and 1A9. Cells were incubated with 0.5×, 1×, 2×, or 4× IC_50_ and incubated at 37 °C for 24 h. DMSO was used as vehicle control. Mean ± SD of three independent measurements.

The generation of reactive oxygen species (ROS) upon exposure of CCRF-CEM cells with *N*-(3-nitrophenylpyrazole) curcumin or 1A9 was compared with the ROS generation by curcumin. As shown in Figure 7, dose-dependent effects were observed with 0.5×, 1×, and 2× IC_50_ concentrations of these three compounds. The strongest ROS generation was measured with 1A9, the lowest one with *N*-(3-nitrophenylpyrazole) curcumin. Curcumin produced intermediate ROS amounts. 

### 2.6. Annexin/PI Assay

**Figure 8:**
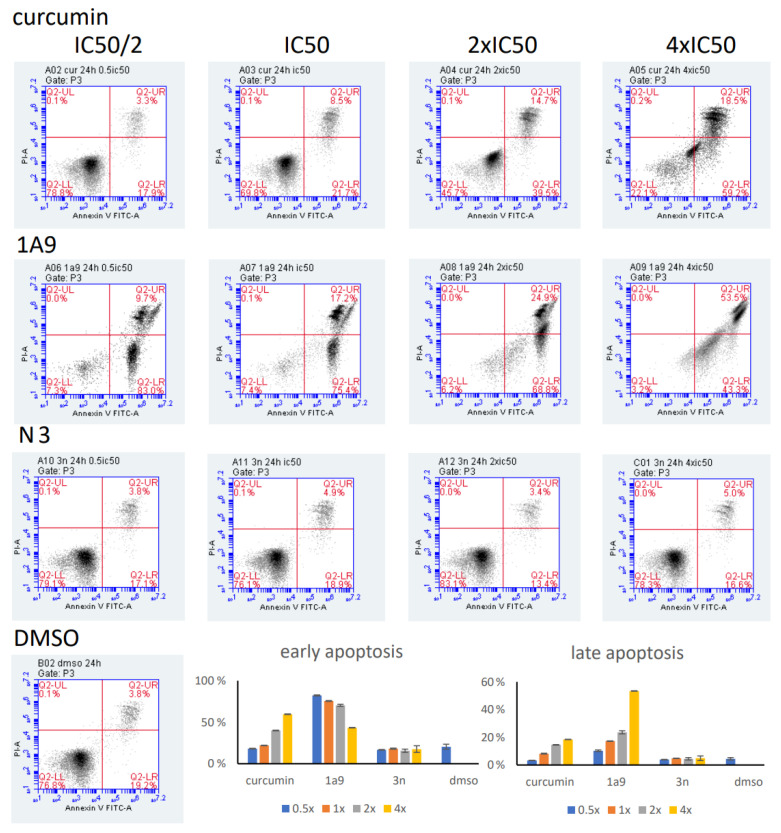
Induction of apoptosis in CCRF-CEM cells upon exposure at 37 °C for 24 h with curcumin, *N*-(3-nitrophenylpyrazole) curcumin, and 1A9 as determined by the annexin V/propidium iodide assay and flow cytometry. Cells were incubated with 0.5×, 1×, 2×, or 4× IC_50_. DMSO was used as vehicle control. Mean ± SD of three independent measurements.

**Figure 9:**
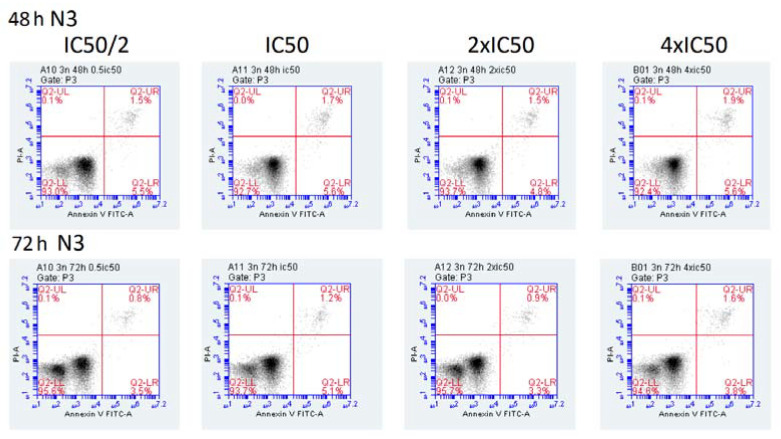

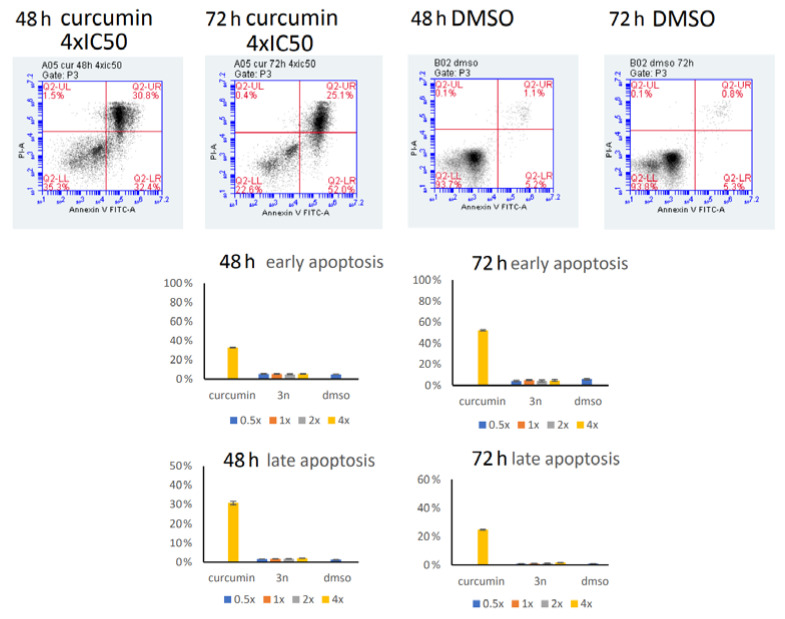
Induction of apoptosis in CCRF-CEM cells upon exposure at 37 °C for 48 or 72 h with *N*-(3-nitrophenylpyrazole) curcumin as determined by the annexin V/propidium iodide assay and flow cytometry. Cells were incubated with 0.5×, 1×, 2×, or 4× IC_50_ *N*-(3-nitrophenylpyrazole) curcumin. Positive control: curcumin (4× IC_50_); negative control: DMSO. Mean ± SD of three independent measurements.

The induction of early and late apoptosis by the three curcumin compounds was determined by the annexin V/propidium iodide assay and flow cytometry. Incubation of CCRF-CEM cells at 37 °C for 24 h showed clear dose-dependent induction either of early or late apoptosis in a concentration range from 0.5× to 4× IC_50_ (Figure 8). The effects of 1A9 were even stronger: the highest fraction of apoptotic cells was observed already at the lowest concentration of 0.5× IC_50_. Higher concentrations led to a decrease in early apoptotic cells and an increase in late apoptotic cells, indicating a concentration-dependent switch from early to late apoptosis upon exposure with 1A9 at 37 °C for 24 h (Figure 8). Although *N*-(3-nitrophenylpyrazole) curcumin was cytotoxic in the LDH assay and inhibited viability in the resazurin assay, it did not induce early or late apoptosis after 24 h incubation (Figure 8). Even after prolonged incubation at 37 °C for 48 or 72 h, *N*-(3-nitrophenylpyrazole) curcumin did not induce apoptosis (Figure 9). The low percentages of apoptosis cells did not exceed the low rates of spontaneous apoptosis in DMSO-treated control cells.

## 3. Discussion

In the present study, we investigated the in silico binding affinities of a total of 50 curcumin compounds to EGFR and NF-κB. The aim was to find novel derivatives with improved binding properties to these two cancer-related proteins as a starting point to develop curcumin-based drugs with improved pharmacological features.

EGFR plays a central role in the pathogenesis and progression of different carcinoma types. EGFR is overexpressed in many human carcinomas and is also involved in developing resistance to chemotherapy [54,56]. On average, 50–70% of lung, colon, and breast carcinoma express EGFR [69]. Inhibition of EGFR phosphorylation is caused by directly inhibitor effects of curcumin on the tyrosine kinase activity of EGFR as well as by curcumin-induced alterations of the physical plasma membrane properties influencing receptor dimerization [70]. Additionally, curcumin downregulated EGFR protein expression [36]. 

From previous investigations on the activity of curcumin derivatives towards EGFR [71], it is known that substitutions on the phenyl rings affected the extent of EGFR down-regulation. Moreover, methoxy or hydroxy substituents increased the compounds’ activity, while other alkyloxy groups did not. The activity was, in general, not influenced by methoxy, hydroxy, or halogen groups. Remarkably, *N*-(3-nitrophenylpyrazole) curcumin was bound better to EGFR than the halogenated curcumin derivatives investigated in this study, including difluorinated curcumin that is frequently described in the literature as a promising drug candidate for further drug development [72].

NF-κB regulates the expression of genes involved in many processes that play a key role in cancer biology, such as proliferation, migration, and apoptosis. NF-κB influences the expression of genes that are involved in a large number of physiological processes, including immune response, cell survival, differentiation, and proliferation. Curcumin has been described as a potent inhibitor of NF-κB activation [73]. Twenty out of 50 curcumin compounds showed binding energies to NF-κB smaller than −10 kcal/mol, while curcumin as a lead compound revealed free binding energies of >−10 kcal/mol. Comparable data were obtained for EGFR: 15 out of 50 curcumin compounds were bound to EGFR with free binding energies of <−10 kcal/mol, while the binding affinity of curcumin itself was >−10 kcal/mol. This indicates that the derivatization of curcumin may indeed be a promising strategy to improve targe specificity and to obtain more effective anticancer drug candidates. 

Even though *N*-(3-nitrophenylpyrazole) curcumin was bound to EGFR and NF- κB, it inhibited cell viability (as shown by the resazurin assay) and was cytotoxic (as shown by the LDH assay) in a comparable manner as curcumin and 1A9, *N*-(3-nitrophenylpyrazole) curcumin did not induce ROS generation and did not induce apoptosis. This indicates that different curcumin derivatives may activate different downstream mechanisms despite identical upstream targets (EGFR, NF-κB). *N*-(3-nitrophenylpyrazole) curcumin apparently induced a ROS-independent non-apoptotic pathway of cell death. This is a novel finding that may have important therapeutic implications. It is well-known that tumor cells with defective apoptotic regulation exert resistance to the induction of cell death, resulting in tumor progression and resistance to anticancer drugs [74,75]. Having cytotoxic drugs at hand, such as *N*-(3-nitrophenylpyrazole) curcumin, may allow one to bypass apoptosis resistance and kill otherwise apoptosis-resistant tumors. It is now well-known that a plethora of different cell death mechanisms can be operative in tumor cells [76,77]. It is beyond the scope of the present investigation to clarify which alternative mechanisms of cell death are responsible for the cytotoxic activity of *N*-(3-nitrophenylpyrazole) curcumin. We will address this issue in the future. 

## 4. Materials and Methods

### 4.1. Chemicals

Chemical structures for in silico analyses were downloaded from PubChem (https://pubchem.ncbi.nlm.nih.gov/; accessed on 31 October 2021). Curcumin and DMSO were of analytical grade and purchased from Sigma-Aldrich (Taufkirchen, Germany). The curcumin derivative 1A9 was synthesized as recently reported [17]. *N*-(3-nitrophenylpyrazole) curcumin was provided by Dr. Fadhil S. Kamounah (Department of Chemistry, University of Copenhagen, Denmark). Stock solutions (50 mM) were prepared in DMSO, stored at −20 °C, and diluted to the final concentration in fresh media before each experiment. The chemical structures of 50 curcumin compounds are shown in Supplementary Figure S1. 

### 4.2. Docking

The X-ray crystallography-based three-dimensional protein structures of EGFR and NF-κB (PDB codes 1M17 and IVKX, respectively) were obtained from the RCSB Protein Data Bank (http://www.rcsb.org/pdb/home/home.do; accessed on 31 October 2021) and used as docking templates throughout the calculations. Two-dimensional structures of curcumin and its derivatives were energy-minimized and converted to 3D structures compatible for docking operation using the Chem 3D program. Molecular docking was then carried out with the AutoDock program 4.2.6 (The Scripps Research Institute, La Jolla, CA, USA) following a previously reported protocol [78,79]. Docking parameters were set to 250 runs and 250,000 energy evaluations for each cycle. Three independent cycles were performed, resulting in a total number of 750,000 calculations per compound and target protein. VMD (Visual Molecular Dynamics, Visual Molecular Dynamics, http://www.ks.uiuc.edu/Research/vmd/; accessed on 31 October 2021) was used as a visualization tool to illustrate further the binding modes obtained from docking. 

### 4.3. Microscale Thermophoresis

In vitro protein binding assays were performed to validate the in silico interaction between EGFR and NF-κB). The recombinant proteins were obtained from commercial sources (EGFR cat. No. 10001-H08H, NF-κB cat. No. 12054-H09E, Sino Biological Europe GmbH, Eschborn, Germany). The proteins were labeled according to the Monolith™ NT.115 Protein Labeling Kit RED-NHS (NanoTemper Technologies GmbH, Munich, Germany). Varying concentrations of curcumin, *N*-(3-phenylpyrazole) curcumin, and the curcumin derivative 1A9 ranging from 100 to 100,000 nM were titrated with labeled EGFR or NF-κB. As previously described, the experiments were carried out using standard capillaries in the NanoTemper Monolith™ NT (NanoTemper Technologies GmbH, Munich, Germany) [80,81]. Microscale thermophoresis (MST) was performed with 40% LED power and 80% MST power for the labeled EGFR and with 20% LED power and 20% MST power for the labeled NF-κB. 

### 4.4. Cell Culture

The A549 lung cancer cell line was obtained from the German Cancer Research Center (DKFZ, Heidelberg, Germany). The original source of the cell lines is the American Type Culture Collection (ATCC, Manassas, VA, USA). The cells were cultivated in a completed DMEM culture medium with GlutaMAX (Invitrogen, Germany) supplemented with 10% fetal bovine serum, l-glutamine (2 mM), and 1% of a 10,000 U/mL penicillin G and 10 mg/mL streptomycin at 37 °C in a humidified air incubator (95%) containing 5% CO_2_. All experiments were performed on cells in the logarithmic growth phase. CCRF-CEM cells were a gift from Dr. Axel Sauerbrey (University Hospital for Pediatrics, Univerity of Jena, Germany) and maintained in an RPMI medium supplemented with fetal bovine serum, glutamine, and penicillin as described above. 

Peripheral blood from healthy donors was obtained from Transfusion Center (University Medical Center, Johannes Gutenberg University Mainz, Mainz, Germany). Human peripheral blood mononuclear cells (PBMCs) were isolated by density gradient centrifugation using Histopaque-1077^®^ (Sigma-Aldrich Co. LLC, Darmstadt, Germany), strictly following the manufacturer’s instructions. Isolated PBMCs were resuspended in warm Panserin 413 medium (PAN-Biotech, Aidenbach, Germany) supplemented with 2% phytohemagglutinin M (PHA-M, Life Technologies, Darmstadt, Germany) and then immediately used for experimentation.

### 4.5. Resazurin Cell Viability Assay

Viable cells reduce the non-fluorescent resazurin to the strongly-fluorescent resorufin. Suspension cells (1 × 10^4^ cells/well) or adherent cells (5 × 10^3^ cells/well, incubated overnight to allow attachment) were seeded in 96-well plates at a volume of 100 µL. Varying concentrations of curcumin, 1A9, or *N*-(3-nitrophenylpyrazole) curcumin were added to reach a total volume of 200 µL. After 72 h, 20 µL of 0.01% *w*/*v* resazurin (Sigma-Aldrich) was added to each well, and cells were incubated for another 4 h at 37 °C. The fluorescence of resorufin was measured at 544 nm (excitation) and 590 nm (emission) using an Infinite M2000 Pro™ plate reader (Tecan, Crailsheim, Germany). Each experiment was independently performed three times with six parallel measurements each. The effects on cell viability were assessed according to the percentage of untreated control and 50% inhibition concentrations (IC_50_) were calculated from dose-response curves using GraphPad Prism^®^ v6.0 software (GraphPad Software Inc., San Diego, CA, USA).

### 4.6. Lactate Dehydrogenase (LDH) Assay

The LDH release from the cells was determined using an LDH-cytotoxicity assay kit (catalog no. ab65393, Abcam, Berlin, Germany). CEM/CCRF leukemia cells and peripheral blood mononuclear cells (PBMCs) were seeded in 96-well plates at a density of 4 × 10^4^ cells/well. Varying concentrations ranging from 0.01 to 10 µM of curcumin, *N*-(3-nitrophenylpyrazole) curcumin, or 1A9 were added to the cells and incubated for 48 h. At the end of the incubation period, the plates were gently shaken to ensure LDH was evenly distributed in the culture medium. Afterward, cells were precipitated at 600× *g* for 10 min and 10 μL/well of the clear supernatant medium were transferred to optically clear 96-well plates. The LDH reaction mix (100 μL/well) was added and incubated for 30 min at room temperature. The absorbance of LDH was measured with an Infinite M2000 Pro™ plate reader (Tecan, Crailsheim, Germany) using a 450 (440–490) nm filter.

### 4.7. Reactive Oxygen Species (ROS) Detection Assay

Intracellular reactive oxygen species (ROS) were determined using the cellular reactive oxygen species detection assay kit (catalog No. ab186027, Abcam, Berlin, Germany). Briefly, 4 × 10^4^ CEM/CCRF leukemia cells/well were seeded in 96-well plates and incubated with 100 µL/well of ROS red working solution for 1 h in an incubator at 37 °C in a 5% CO_2_ atmosphere. Afterward, cells were treated with curcumin, *N*-(3-nitrophenylpyrazole) curcumin, or 1A9. For each compound, varying concentrations (0.5× IC_50_, IC_50_, 2× IC_50_) were used. After incubation for 24 h, the ROS induction was monitored at Ex/Em = 520/605 nm using an Infinite M2000 Pro™ plate reader (Tecan, Crailsheim, Germany). 

### 4.8. Annexin V/Propidium Iodide (PI) Assay

CCRF-CEM cells (1 × 10^6^ cells/mL) were seeded in 6-well plates and different concentrations of curcumin, 1A9, or *N*-(3-nitrophenylpyrazole) curcumin (0.5×, 1×, 2×, 4× IC_50_) were applied at 37 °C for 24 h. The cells were washed and re-suspended in 1 mL cold PBS and 500 μL 1× binding buffer, respectively, followed by incubation with 5 µL annexin V/PI and 10 µL of PE (50 mg/mL) (Thermo Fisher Scientific, Dreieich, Germany) in the dark for 15 min. The histograms were measured using an Accuri C6 flow cytometer (Becton Dickinson, Heidelberg, Germany). Four different cell populations were determined: viable cells, annexin V positive/PI negative cells (cells that are in early apoptosis), annexin V negative/PI-positive cells (cells that are necrotic), and annexin V positive/PI-positive cells (cells that are in late apoptosis or already dead).

## 5. Conclusions

In conclusion, we speculated that synthetic derivatives bear potential for cancer treatment, especially as EGFR inhibitors. Some synthetic derivatives are bound to and inhibit their target proteins in a comparable or even better manner than their parent lead compound, curcumin. The chemistry of curcumins is a fascinating area of research. It should be kept in mind that curcumin is not only active against cancer but also against many diseases such as metabolic syndrome, ulcerative colitis and inflammatory diseases in general, neurodegenerative diseases, infectious diseases, etc. [82,83,84,85,86]. It is well in the scope of expectations that some of the curcumin derivatives described in the present study may also be valuable drug candidates for other diseases than cancer. We believe they may be valuable candidates in the further drug discovery process.

## Figures and Tables

**Table 1:** Mean binding energies and predicted binding constants were obtained by molecular docking for 50 curcumin compounds. Dockings were independently carried out three times with every 250,000 runs (mean values ± SD).

Compounds		EGFR (Mean Binding Energy, kcal/mol)	EGFR (Mean pKi, µM)	NF-κB (Mean Binding Energy, kcal/mol)	NF-κB (Mean pKi, µM)
1	Curcumin	−9.42 ± 0.09	0.13 ± 0.02	−8.38 ± 0.16	0.73 ± 0.21
2	Bisdemethoxycurcumin	−8.51 ± 0.02	0.58 ± 0.02	−7.96 ± 0.05	1.46 ± 0.11
3	Diacetylcurcumin	−10.72 ± 0.49	0.02 ± 0.02	−9.67 ± 0.26	0.09 ± 0.04
4	[^18^FP]-curcumin	−8.70 ± 0.21	0.43 ± 0.14	−9.01 ± 0.07	0.25 ± 0.03
5	Monodemethoxycurcumin	−9.04 ± 0.06	0.24 ± 0.02	−8.07 ± 0.10	1.23 ± 0.20
6	(E,E)-Bis(2-hydroxybenzylidene)acetone	−7.95 ± 0.01	1.49 ± 0.04	−7.21 ± 0.06	5.19 ± 0.50
7	Curcumin monoglucoside	−9.72 ± 0.76	0.11 ± 0.11	−9.57 ± 0.34	0.11 ± 0.06
8	Di-*O*-(2-thienoyl) curcumin	−11.71 ± 0.26	0.003 ± 0.002	−10.71 ± 0.17	0.01 ± <0.01
9	Cis-curcumin	−9.07 ± 0.30	0.24 ± 0.10	−8.81 ± 0.33	0.39 ± 0.21
10	Curcumin diglucoside	−9.10 ± 0.99	0.39 ± 0.35	−10.35 ± 0.35	0.03 ± 0.02
11	Tetrahydrocurcumin	−9.13 ± 0.17	0.21 ± 0.06	−8.28 ± 0.21	0.90 ± 0.34
12	Allyl curcumin	−10.07 ± 0.07	0.04 ± 0.01	−9.15 ± 0.04	0.20 ± 0.01
13	Monodemethylcurcumin	−9.96 ± 0.10	0.05 ± 0.01	−8.16 ± 0.56	0.59 ± 0.12
14	Didemethylcurcumin	−9.90 ± 0.07	0.06 ± 0.01	−8.71 ± 0.25	0.49 ± 0.18
15	Curcumin-4′-*O*-β-d-gentiotrioside	−7.85 ± 0,59	3.11 ± 0.23	−7.51 ± 0.37	3.47 ± 2.05
16	4-Benzylidene curcumin	−10.62 ± 0.72	0.03 ± 0.03	−9.10 ± 0.10	0.21 ± 0.04
17	Monoglycinoyl curcumin	−12.12 ± 0.21	0.0013 ± 0.0006	−10.07 ± 0.08	0.04 ± 0.01
18	Ethyl curcumin	−10.09 ± 0.11	0.04 ± 0.01	−9.23 ± 0.05	0.17 ± 0.01
19	Curcumin dimer 1	−11.64 ± 0.56	0.004 ± 0.003	−11.11 ± 0.65	0.01 ± 0.01
20	*N*-phenylpyrazole curcumin	−8.63 ± 0.19	0.49 ± 0.14	−8.81 ± 0.01	0.35 ± 0.01
21	Curcumin β-d-glucuronide	−10.36 ± 0.19	0.04 ± 0.02	−10.07 ± 0.36	0.05 ± 0.03
22	3,4-Difluorobenzylidene curcumin	−10.08 ± 0.13	0.04 ± 0.01	−9.30 ± 0.07	0.15 ± 0.02
23	Di-*O*-(2-hydroxyethyl) curcumin	−9.60 ± 0.27	0.10 ± 0.05	−8.97 ± 0.55	0.35 ± 0.27
24	4-(4-hydroxybenzylidene) curcumin	−10.55 ± 0.12	0.02 ± <0.01	−8.98 ± 0.04	0.26 ± 0.02
25	*N*-(3-nitrophenylpyrazole) curcumin	−10.50 ± 0.02	0.02 ± <0.01	−9.57 ± 0.01	0.097 ± <0.01
26	*N*-(4-flurophenylpyrazole) curcumin	−8.46 ± 0.19	0.65 ± 0.19	−8.81 ± 0.09	0.35 ± 0.06
27	*N*-(4-methoxyphenylpyrazole) curcumin	−8.49 ± 0.04	0.60 ± 0.03	−8.95 ± 0.11	0.28 ± 0.05
28	Di-*O*-chloropropionylethyl curcumin	−10.92 ± 0.25	0.01 ± <0.01	−10.36 ± 0.59	0.03 ± 0.02
29	Curcumin tri-adamantylaniniethylcarbonate	−10.75 ± 0.23	0.01 ± 0.01	−12.97 ± 0.47	0.0004 ± 0.0003
30	Curcumin dimer 2	−11.08 ± 0.24	0.01 ± <0.01	−12.05 ± 0.46	0.002 ± 0.001
31	Curcumin tri-trithiadiazolaminoethylcarbonate	−10.35 ± 0.55	0.03 ± 0.02	−10.97 ± 0.53	0.01 ± 0.01
32	4-(4-hydroxy-3-methoxybenzylidene) curcumin	−11.44 ± 0.16	0.004 ± 0.002	−10.97 ± 0.53	0.01 ± 0.01
33	Curcumin-β-d-glucuronide triacetate methyl ester	−10.06 ± 0.79	0.07 ± 0.06	−10.35 ± 0.35	0.03 ± 0.02
34	Curcumin 4′-*O*-β-d-gentiobiosyl 4″-*O*-β-d-glucoside	−9.54 ± 0.25	0.11 ± 0.05	−10.23 ± 0.59	0.04 ± 0.04
35	Tetrahydrocurcumin isoxazole	−9.43 ± 0.20	0.13 ± 0.04	−8.60 ± 0.41	0.57 ± 0.32
36	Hexahydrocurcumin	−9.25 ± 0.11	0.17 ± 0.03	−8.41 ± 0.27	0.73 ± 0.35
37	Bisdemethoxycurcumin isoxazole	−7.45 ± 0.01	3.45 ± 0.10	−7.55 ± 0.10	2.96 ± 0.47
38	Curcumin dimer 3	−10.28 ± 0.22	0.03 ± 0.01	−10.50 ± 0.77	0.01 ± 0.01
39	Curcumin ED	−9.59 ± 0.08	0.10 ± 0.02	−8.69 ± 0.14	0.43 ± 0.10
40	Curcumin PE	−9.24 ± 0.12	0.17 ± 0.03	−8.22 ± 0.22	0.99 ± 0.33
41	Curcumin sulfate	−9.31 ± 0.16	0.15 ± 0.04	−9.84 ± 0.08	0.06 ± 0.01
42	Ferrocenyl curcumin	−8.34 ± 0.22	0.80 ± 0.29	−7.10 ± 0.33	6.87 ± 3.92
43	Di-*O*-deconyl curcumin	−10.57 ± 0.17	0.01 ± <0.01	−11.58 ± 0.85	0.02 ± 0.01
44	GNF-pf-2695 ((2E,5E)-2,5-bis[(3,4,5-trimethoxyphenyl) methylidene]cyclopentan-1-one)	−7.34 ± 0.07	4.22 ± 0.51	−7.56 ± 0.15	2.93 ± 0.78
45	Perfluoro curcumin	−7.55 ± 0.19	3.03 ± 0.86	−6.86 ± 0.28	10.05 ± 4.03
46	Keto-curcumin	−9.89 ± 0.41	0.07 ± 0.04	−8.78 ± 0.15	0.37 ± 0.09
47	Disalicyloyl curcumin	−7.67 ± 0.58	0.02 ± 0.02	−6.24 ± 0.06	26.77 ± 3.36
48	HO-3867 (3E,5E)-3,5-bis[(4-fluorophenyl)methylidene]-1-[(1-hydroxy-2,2,5,5-tetramethylpyrrol-3-yl)methyl]piperidin-4-one)	−9.73 ± 0.03	0.07 ± <0.01	−7.79 ± 0.10	1.96 ± 0.33
49	1A6 ((1E,6E)-4-chloro-1,7-bis(3,4-dimethoxyphenyl)hepta-1,6-diene-3,5-dione)	−8.94 ± 0.06	0.28 ± 0.03	−8.28 ± 0.21	0.90 ± 0.34
50	1A9 ((1E,6E)-4-chloro-1,7-di(1H-indol-3-yl)hepta-1,6-diene-3,5-dione)	−9.57 ± 0.02	0.10 ± 0.03	−8.51 ± 0.11	0.58 ± <0.01

**Table 2:** IC_50_ values of CCRF-CEM leukemia and A549 lung carcinoma cells treated with curcumin, 1A9, and *N*-(3-nitrophenylpyrazole) curcumin as determined by the resazurin assay. The IC_50_ values (µM) were calculated from the dose-response curves shown in Figure 5. DMSO was used as vehicle control. Mean ± SD of three independent measurements.

Cells		Compounds	
	Curcumin	*N*-(3-Nitrophenylpyrazole) Curcumin	1A9
CCRF-CEM	3.0 ± 0.4	1.9 ± 0.4	2.6 ± 0.3
A549	29.4 ± 1.9	18.9 ± 1.4	23.3 ± 1.2
PBMC	>100	>100	>100

## Data Availability

Data available on request.

## Supplementary Materials

Supplementary materials can be found at https://www.mdpi.com/article/10.3390/ijms23073966/s1.
