# Supplementary material for: In Silico and In Vitro Screening of 50 Curcumin Compounds as EGFR and NF-κB Inhibitors
Source: Int J Mol Sci. 2022 Apr 2;23(7):3966. doi: 10.3390/ijms23073966 (PMC9000198; doi:10.3390/ijms23073966)
Supplement: Supplementary file 1 [file ijms-23-03966-s001.zip › ijms-1421225-supplementary.pdf]

Supplementary Figure S1

In silico and *in vitro* drug screening of curcumin and derivatives as EGFR and NF-κB inhibitors

Mohamed E. M. Saeed[1], Rümeysa Yücer[1,2], Mona Dawood[1,3], Mohamed-Elamir F. Hegazy[1,4], Assia Drif[1], Edna Ooko[1], Onat Kadioglu[1], Ean-Jeong Seo[1], Fadhil S. Kamounah[5], Salam J. Titinchi[6], Beatrice Bachmeier[7], Thomas Efferth[1*]

[1] Department of Pharmaceutical Biology, Institute of Pharmacy and Biochemistry, Johannes Gutenberg University, Staudinger Weg 5, 55128, Mainz, Germany
[2] home addresses: Department of Pharmacognosy, Hamidiye Faculty of Pharmacy, University of Health Sciences Turkey, Üsküdar 34668 İstanbul, Turkey; and Department of Pharmacognosy and Phytochemistry, Faculty of Pharmacy, Bezmialem Vakif University, 34093 Istanbul, Turkey
[3] home address: Faculty of Medical Laboratory Sciences, Al-Neelain University, Khartoum, Sudan
[4] home address: Chemistry of Medicinal Plants Department, National Research Centre, 33 El-Bohouth St., Dokki, Giza 12622, Egypt.
[5] Department of Chemistry, University of Copenhagen, Universitetsparken 5, Copenhagen 2100, Denmark
[6] Department of Chemistry, University of the Western Cape, P/B X17, Bellville, 7535 Cape Town, South Africa.
[7] Institute of Pharmaceutical Biology, Goethe University, Frankfurt/Main, Germany
* Corresponding author: Tel: +49-6131-39-25751. Fax: +49-6131-39-23752. E-mail: efferth@uni-mainz.de

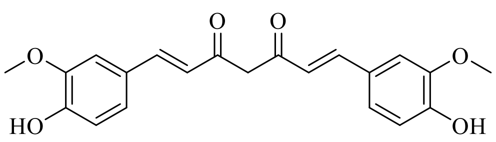

curcumin

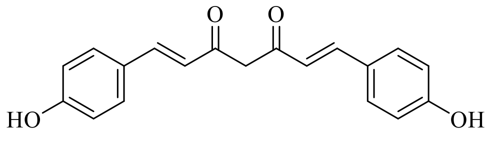

Bisdemethoxycurcumin

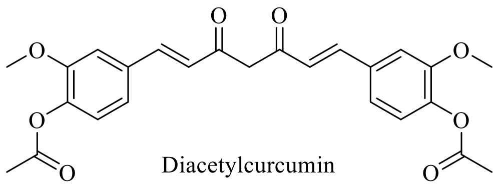

Diacetylcurcumin

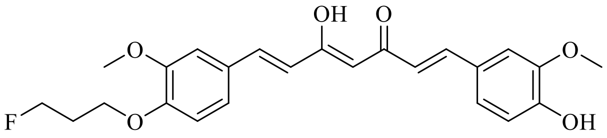

[18FP]-curcumin

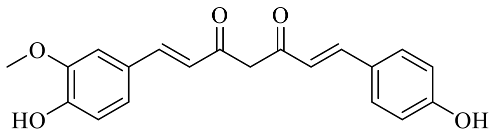

Monodemethoxycurcumin

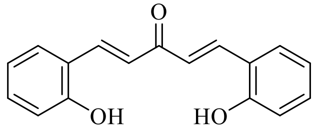

(E,E)-Bis(2-hydroxybenzylidene)acetone

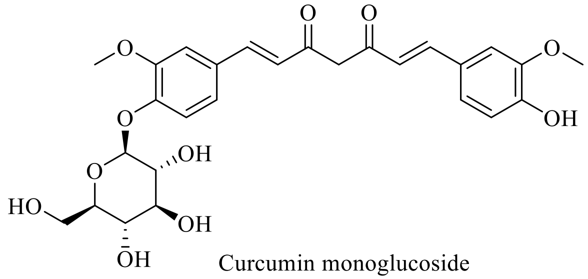

Curcumin monoglucoside

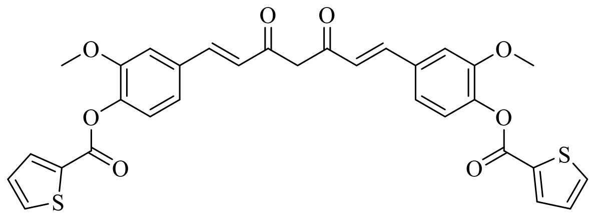

Di-*O*-(2-Thienoyl) curcumin

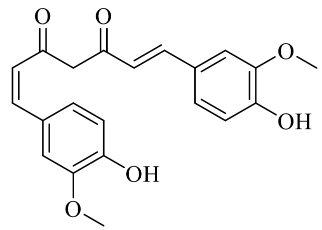

Cis-curcumin

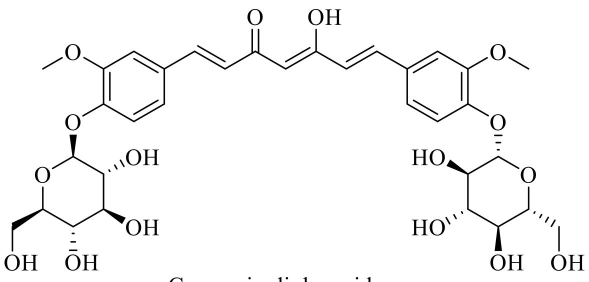

Curcumin diglucoside

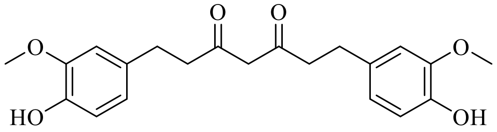

Tetrahydrocurcumin

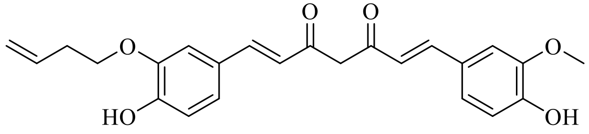

Allyl curcumin

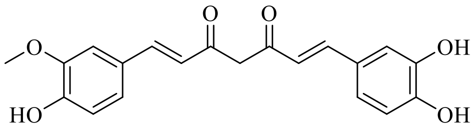

Monodemethylcurcumin

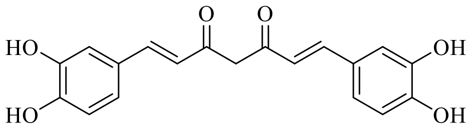

Didemethylcurcumin

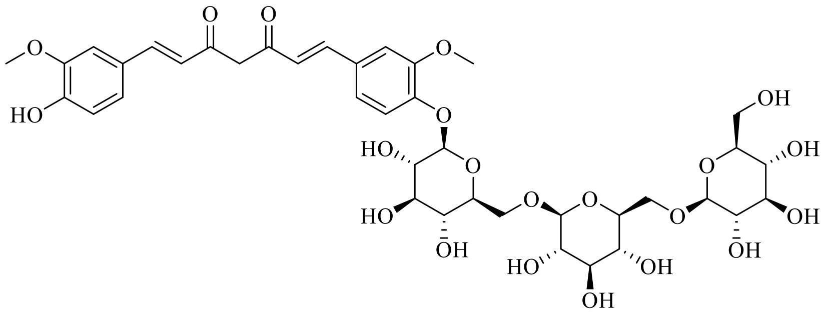

Curcumin-4'-*O*-β-*D*-gentiotrioside

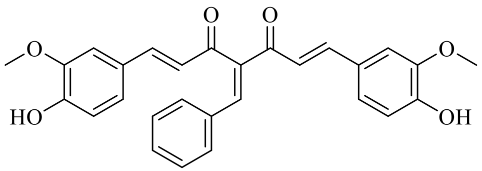

4-Benzylidene curcumin

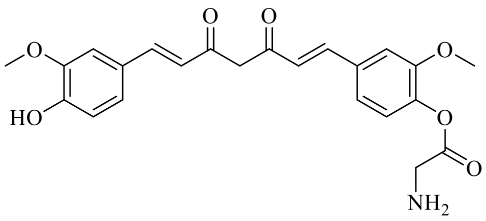

Monoglycinoyl curcumin

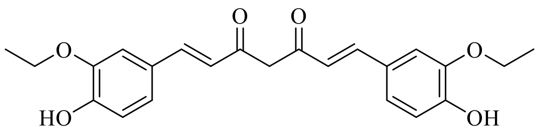

Ethylcurcumin

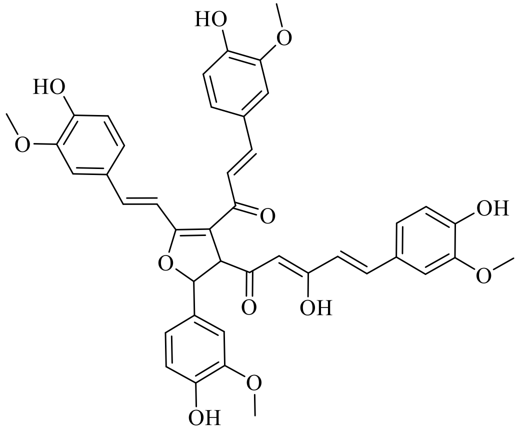

Curcumin dimer 1

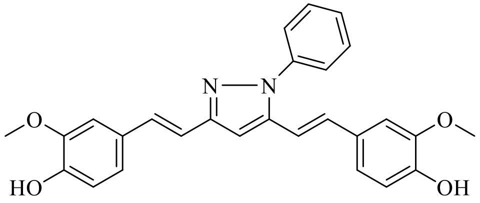

N-phenylpyrazole curcumin

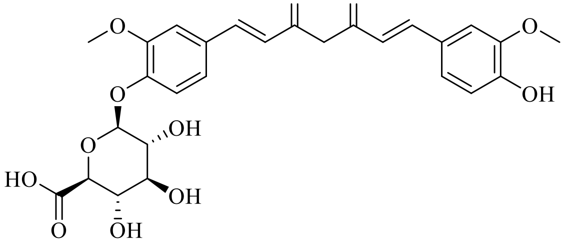

Curcumin β-*D*-glucuronide

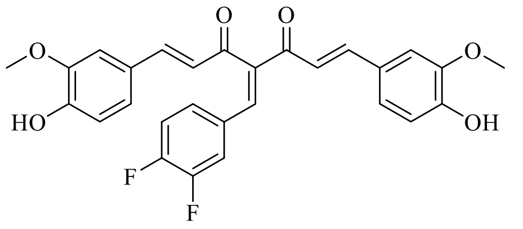

3,4-Difluorobenzylidene curcumin

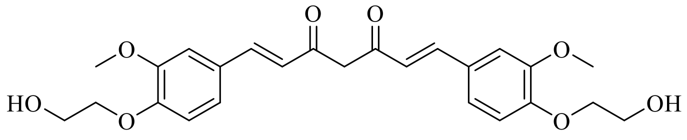

Di-*O*-(2-hydroxyethyl) curcumin

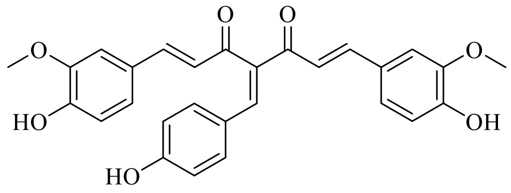

4-(4-hydroxybenzylidene) curcumin

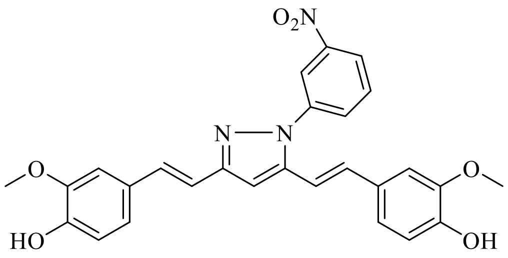

N-(3-nitrophenylpyrazole) curcumin

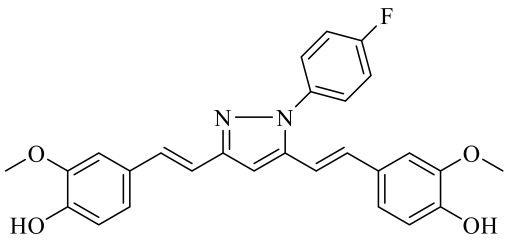

N-(4-flurophenylpyrazole) curcumin

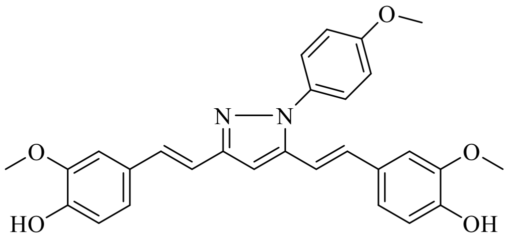

N-(4-methoxyphenylpyrazole) curcumin

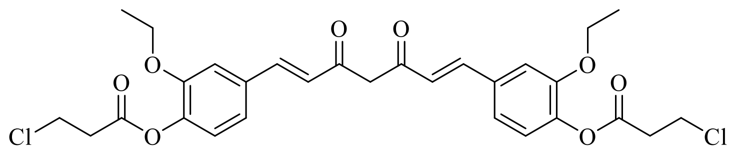

Di-*O*-chloropropionylethyl curcumin

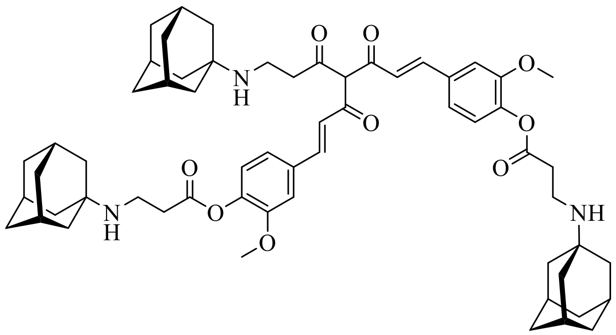

Curcumin tri adamantylaminoethylcarbonate

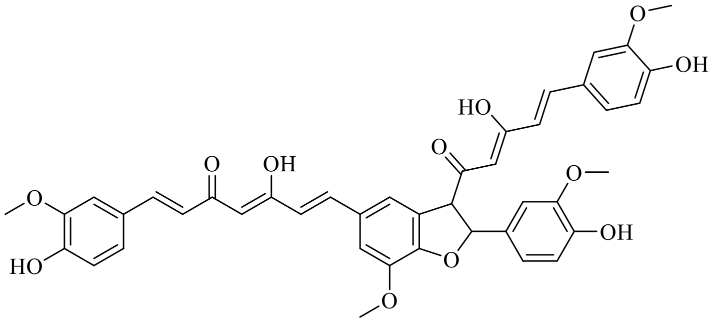

Curcumin dimer 2

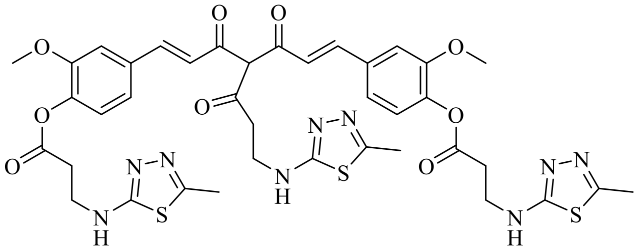

Curcumin tri-trithiadiazolaminoethylcarbonate

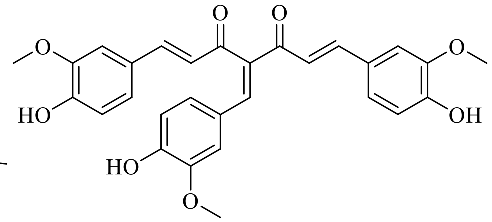

4-(4-hydroxy-3-methoxybenzylidene) curcumin

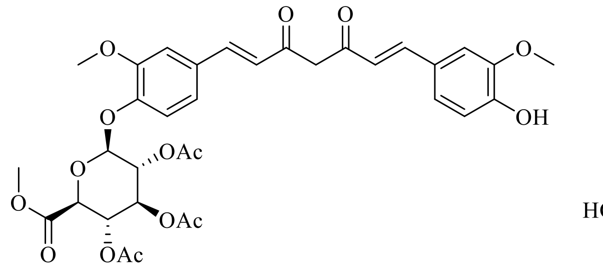

Curcumin β-*D*-glucuronide triacetate methyl ester

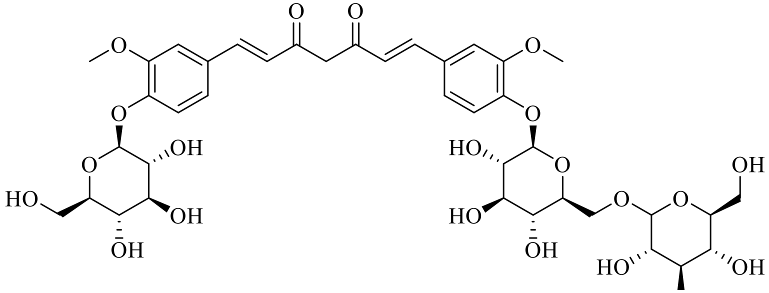

Curcumin 4'-*O*-β-D-gentiobiosyl 4''-*O*-β-D-glucoside

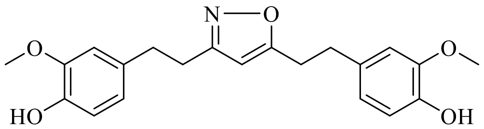

Tetrahydrocurcumin isoxazole

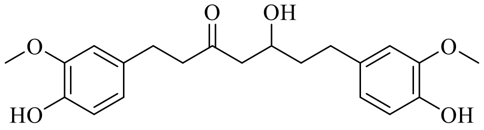

Hexahydrocurcumin

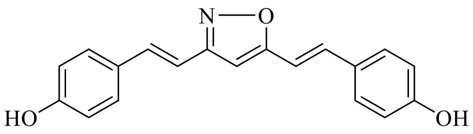

Bisdemethoxycurcumin isoxazole

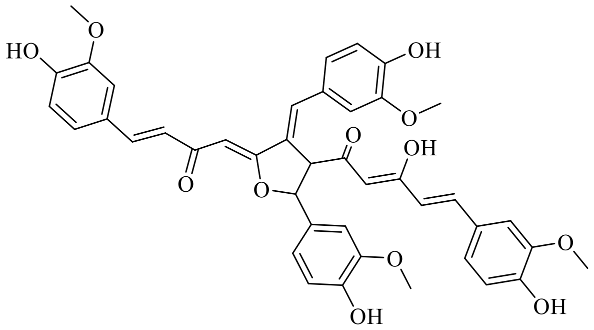

Curcumin dimer 3

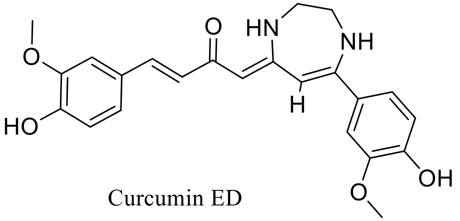

Curcumin ED

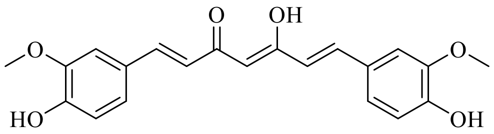

Curcumin PE

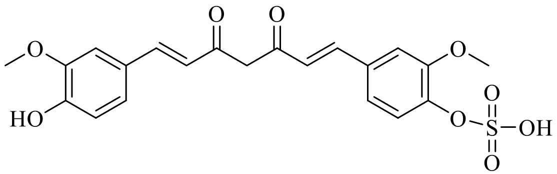

Curcumin sulfate

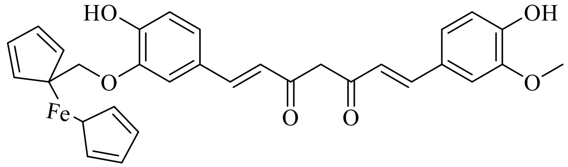

Ferrocenyl curcumin

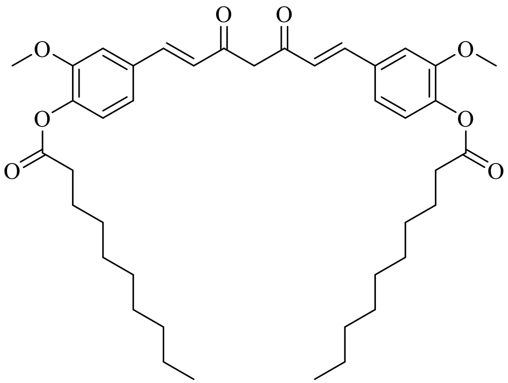

Di-*O*-decanoyl curcumin

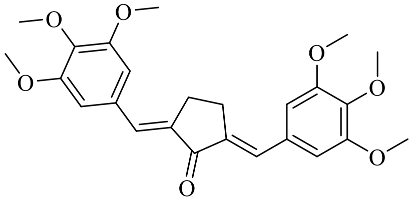

GNF-pf-2695

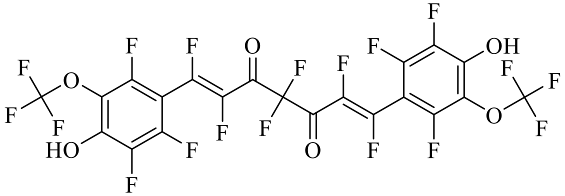

Perfluoro curcumin

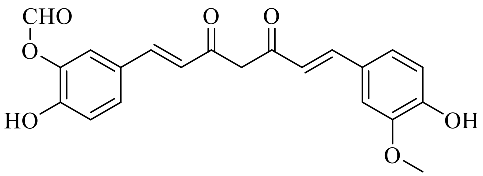

Keto-curcumin

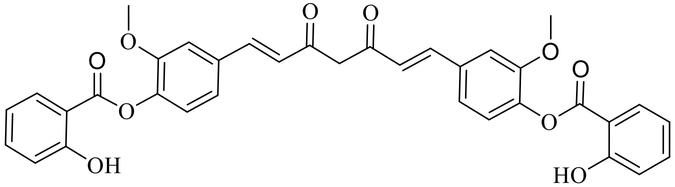

Disalicyloyl curcumin

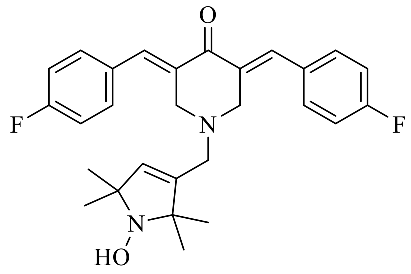

HO-3867

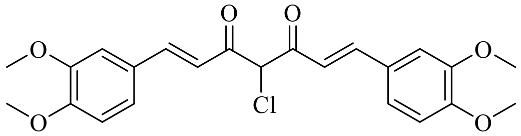

1A6

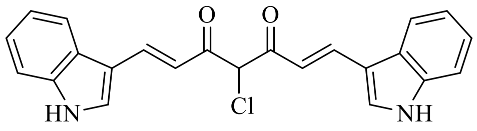

1A9
